# 

**Published:** 1912-03

**Authors:** 


VOL. XXVII — MARCH, 1912 — No. 9

### TEXAS MEDICAL JOURNAL

Established July, 1885

*"Red Back"*

PUBLISHED MONTHLY AT AUSTIN, TEXAS, BY

F. E. DANIEL, M. D., - - - Editor and Proprietor

PRINTED BY THE VON BOECKMANN-JONES [illegible]

Subscription, $1.00 a Year, in Advance. - - - - Single Copies, 10 Cents

Entered at the Postoffice at Austin, Texas, as second class mail matter.

